# Microbiota–Metabolome Alterations Suggest Pro‐Inflammatory Phenotype in Congenital Central Hypoventilation Syndrome

**DOI:** 10.1155/ijm/7551500

**Published:** 2026-09-07

**Authors:** Leandro Di Gloria, Marta Peruzzi, Marzia Vasarri, Marta Menicatti, Gianluca Bartolucci, Monica Annunziata, Arianna Bedeschi, Matteo Ramazzotti, Donatella Degl’Innocenti, Niccolò Nassi

**Affiliations:** ^1^ Department of Experimental and Clinical Biomedical Sciences, University of Florence, Florence, Italy, unifi.it; ^2^ Sleep Disorder Breathing and SIDS Center, Meyer Children’s Hospital IRCCS, Florence, Italy; ^3^ Department of Neurosciences, Psychology, Drug Research and Child Health Section of Pharmaceutical and Nutraceutical Sciences, University of Florence, Florence, Italy, unifi.it; ^4^ Gastroenterology Unit, Meyer Children’s Hospital IRCCS, Florence, Italy

**Keywords:** CCHS, dysbiosis, inflammation, metabolic profiling, microbiota

## Abstract

Congenital central hypoventilation syndrome (CCHS) is a genetic disorder caused by mutations in the PHOX2B gene, characterized by impaired autonomic control of breathing and systemic consequences that may affect gut homeostasis. This study provides the first integrated multi‐omics analysis in a CCHS group and matched controls, combining fecal DNA‐based gut taxonomic profiling with targeted quantification of fatty acids and aromatic carboxylic acids. While overall microbial diversity and community structure remained largely preserved, significant alterations were observed in specific taxa within the CCHS group. Notably, the control group exhibited an enrichment of short‐chain fatty acid (SCFA)‐producing genera, which are associated with eubiotic gut ecosystems, whereas the CCHS group showed higher abundance of taxa commonly linked to inflammatory contexts. Consistently, fecal levels of beneficial SCFAs—particularly valeric acid, and to a lesser extent butyric acid—were reduced in CCHS group. These findings point to a dysbiotic gut microbiota in patients with CCHS, likely supporting putative inflammatory processes that would further worsen overall health status if confirmed. Furthermore, this work provides exploratory functional signatures for future studies aimed at understanding systemic consequences, guiding mechanistic investigations, and informing strategies to improve supportive care and long‐term health outcomes in this rare patient population.

## 1. Introduction

Autonomous physiological functions such as breathing, heart rate regulation, and gastrointestinal motility constitute the fundamental biological processes underlying human life and operate in the background of conscious experience, even as the reader breathes without deliberate awareness. These processes are governed by the autonomic nervous system (ANS), which maintains homeostasis through involuntary neural regulation. In individuals with congenital central hypoventilation syndrome (CCHS), this regulation is severely impaired due to a rare, life‐threatening genetic disorder. CCHS affects approximately 1 in 200,000 live births and is caused by heterozygous mutations in the PHOX2B (paired‐like homeobox 2b) gene, a transcription factor critical for the proper development of the ANS and neural crest‐derived structures [1]. CCHS is characterized by an impaired autonomic control of breathing, particularly during sleep, resulting in chronic hypoventilation, apnea, and hypopnea [2, 3]; therefore, ventilatory support is mandatory at least during sleep. This respiratory dysfunction is directly linked to altered development of brainstem and peripheral autonomic circuits caused by PHOX2B mutations. However, CCHS is increasingly recognized as a multisystemic disorder with frequent co‐occurrence of intestinal aganglionosis (such as Hirschsprung disease) and an elevated risk of malignant tumors arising from neural crest cells (including neuroblastoma, ganglioneuroma, and ganglioneuroblastoma). These diverse manifestations reflect the broader developmental role of PHOX2B beyond respiratory regulation [1].

Recent studies have highlighted a potential role for oxidative stress in CCHS. High levels of reactive oxygen species (ROS) have been observed in affected individuals, suggesting that redox imbalance may contribute to immune dysregulation, systemic inflammation, and cellular damage [4, 5]. Although CCHS is a lifelong disorder with no curative treatment due to its genetic basis, the increasing survival into adulthood compared with the predominantly pediatric prognosis observed in the past underscores the need for optimized long‐term care and secondary prevention strategies aimed at improving patients’ quality of life [6, 7].

Increasing attention is being paid to the gut–brain axis and the role of the intestinal microbiota in modulating inflammation and systemic health. Notably, PHOX2B mutations affect not only central autonomic regulation but also the enteric nervous system, leading to conditions such as Hirschsprung disease in roughly about 16% of CCHS cases. This disruption of neural control within the gut may result in abnormal intestinal absorption, chronic constipation, and local inflammation, potentially contributing to gut microbiota dysbiosis. Given the role of the enteric nervous system in regulating intestinal motility and local immune responses, it is plausible that CCHS may be associated with shifts in gut microbiota. Dysbiosis, defined as an imbalance in the resident gut microbial community, has been implicated in a wide range of conditions, from neurodevelopmental disorders to metabolic and inflammatory diseases. In this context, addressing potential gut microbiota dysbiosis may prove important, as the gut microbiota is now recognized as a functional endocrine organ [8]. Indeed, the gut microbiota is a dynamic ecosystem of bacteria, fungi, viruses, and archaea living in mutualistic symbiosis with the host. Collectively, this microbial community constitute a genetic pool of 3 million genes (150 times larger than that of the human genome) [9] performing a wide array of essential functions, including, but not limited to, positively modulating the activity of the gut‐associated lymphoid tissue, stimulating the proliferation of lymphocytes [10] and fermenting nutrients, such as dietary fibers, which are indigestible by human enzymes [11]. Metabolites such as fatty acids (FAs) and aromatic carboxylic acids (ACAs) are obtained from such fermentations. Notably, the gut microbiota represents the primary source of various FAs and ACAs involved in human metabolism [11–13], although small amounts of FAs, excluding few “essential” long‐chain fatty acids (LCFA) and certain ACAs, can also be produced endogenously by the host [13–16] or be directly assimilated from foods such as milk and vegetal oils [17, 18]. For example, while most of the short‐chain fatty acids (SCFAs) are derived from bacterial fermentation, trace quantities are synthesized endogenously in the human liver and adipose tissues [14, 16]. Nevertheless, the biosynthesis by gut bacteria remains the main source especially of SCFAs [12, 16, 19] and ACAs [13, 19–22], with a less predominant contribution on medium‐chain fatty acids (MCFA) and LCFA quantity [16]. Although most of produced SCFAs are consumed by the bacteria themselves, the remaining fraction is metabolized by the enterocytes, with only a small portion entering systemic circulation [11, 12, 16, 23]. The SCFAs are considered among the most important metabolites from the gut microbiota as they not only serve as energy source but also play a crucial role in regulating numerous pathways associated with the immune system, energy metabolism, epigenetic regulation, modulation of inflammation, and increased levels of the antioxidant enzyme superoxide dismutase (SOD) [10–12, 14, 24–26]. Furthermore, FAs are able to cross the blood–brain barrier, with the SCFAs in particular playing a key role in the gut–brain communication axis by exerting neuroactive properties [11, 12, 18, 27]. Recent articles have suggested that certain ACAs may cross the barrier as well [21]. In addition, recently, the microbiota has been implicated in hypotheses regarding the lung–gut axis modulation; thus, the microbiota is speculated to influence respiratory diseases [28]. Accordingly, profiling gut microbial metabolites such as SCFAs and ACAs may provide additional insight into functional alterations of the microbiota and their potential contribution to inflammation and redox balance in patients with CCHS.

Both diet and host genetics play a crucial role in shaping the intestinal microbiota, influencing which microbial taxa can thrive as well as which pathway they exert [12, 29], thus effecting the metabolites production. Despite the potential impact outlined above, as far as we know, scientific insights into the microbiota of patients with CCHS are not available in current literature. Accordingly, this research focuses on profiling the microbiota of individuals affected by CCHS compared with control group using their fecal samples as a proxy to minimize the invasiveness of the study, while analyzing fecal FA and ACA levels to estimate the involvement in inflammation and ROS modulation.

In a rare and genetically defined disorder such as CCHS, understanding secondary and potentially modifiable biological processes is essential for improving long‐term patient management. While the genetic basis of CCHS is well established, the systemic consequences of the syndrome on host–microbe interactions and gut metabolic function remain largely uncharacterized. Studies profiling gut microbiota in rare genetic diseases with a defined causative gene are extremely limited; among such conditions, only cystic fibrosis and CDKL5 deficiency disorder have been explored, making comparative data scarce. Emerging evidence suggests that gut‐derived metabolites, such as SCFAs and ACAs, may influence systemic processes through the gut–brain and gut–lung axes, potentially modulating neuroimmune and respiratory functions. In CCHS, where PHOX2B mutations affect both central and enteric autonomic circuits, profiling the gut microbiota and its metabolic output could provide insights into these systemic interactions. This underscores the novelty and significance of investigating gut microbiota and metabolite profiles in CCHS. Assessing gut microbiota composition and associated metabolic outputs through an integrated multi‐omics approach combining metagenomics and metabolomics can provide functional insights into downstream inflammatory and metabolic alterations. Although not aimed at identifying immediately translatable clinical biomarkers, this approach may inform future mechanistic studies and support the development of personalized supportive strategies, including nutritional interventions and microbiota‐targeted approaches, to help maintain systemic homeostasis and improve long‐term health outcomes in individuals with CCHS.

## 2. Materials and Methods

### 2.1. Participant Sampling and Recruitment: CCHS and Control Groups

Participants were recruited to include both individuals diagnosed with CCHS and control subjects. All participants were of Italian nationality and followed an omnivorous diet. Patient information, including age and possible comorbidities (e.g., Hirschsprung disease), was collected and evaluated for potential influence on the obtained results (Table S1). The study included individuals aged between 9 and 52 years (with only two subjects younger than 12, one in each group), following an omnivore diet and not assuming pharmaceutical drugs in the week of the sample collection. Only one CCHS sample (labeled as “C1”) was taking a lactose‐based prebiotic supplement.

Inclusion criteria for patients with CCHS were a confirmed genetic diagnosis of PHOX2B mutation, age ≥ 9years, ability to provide a stool sample, and the absence of acute illness at the time of sample collection. Exclusion criteria included gastrointestinal disorders unrelated to CCHS, recent antibiotic therapy (within the past month) or pregnancy. For controls, inclusion criteria were the absence of chronic diseases, the absence of gastrointestinal disorders, and similar age range, while exclusion criteria mirrored those of the CCHS group.

Given the rarity of patients with CCHS, samples were enrolled in the study nevertheless, and any potential biases related to its inclusion were carefully evaluated during data interpretation. Moreover, it is noteworthy that this study was specifically designed to prioritize the recruitment of participants from the same household whenever feasible. As a result, approximately half of the control subjects were parents of the patients with CCHS. Although this approach helps mitigate the bias introduced by differences in household environments [30], it also led to a slightly higher average age in the control group (mean 35, range 9–51) compared with the CCHS group (mean 24, range 11–40). To address this, we carefully verified that the observed differences between groups were not driven by age, through both statistical and biological evaluations.

Ethical approval for this study was obtained from Ethics Committee of the Region of Tuscany – Pediatrics, May 3, 2024, no 57/2024, and all participants or their legal guardians provided written informed consent in accordance with the Declaration of Helsinki.

Table S1 provides the characteristics of all enrolled participants, including age, sex, comorbidities, household membership, and any relevant supplementation, as well as any sample replicates used in the preliminary technical overview of the dataset.

### 2.2. Fecal Sample Collection

Fecal samples were collected from 18 patients with CCHS and 17 control subjects and stored at −80°C until processing. Notably, control relatives of the patients CCHS were recruited, resulting in 8 out of 17 control subjects being family members of patients with CCHS. For the purposes of this study, relatives were referred to as “belonging to the same household”. Moreover, fecal samples from one control subject and two patients with CCHS were collected approximately 1 month after the initial sampling, to serve as biological replicates for estimating the individual microbiota variability compared with the inter‐group variation.

### 2.3. 16S DNA Library Preparation

The DNeasy PowerFecal Pro Kit (Qiagen, Hilden, Germany) was used to extract the total DNA from the frozen feces, following the manufacturer’s instructions. Quality and quantity of extracted DNA were assessed with NanoDrop ONE (Thermo Fisher Scientific, Waltham, United States). Afterwards, 12.5 ng of DNA were used as input in 25 PCR cycles according to Illumina 16S library preparation protocol, using the Platinum SuperFi II kit (Invitrogen, Massachusetts, United States). In particular, the DNA template encompassing the region V3–V4 of the 16S rRNA gene was amplified using the primers Pro341FB (CCTACGGGNBGCWSCAG) and 805R (GACTACHVGGGTATCTAATCC) [31]. Afterwards, amplicons from each sample were normalized to the same concentration and sent to BMR Genomics S.r.l. (Padova, Italy) where it was sequenced in paired‐end mode (2 × 300 *c* *y* *c* *l* *e* *s*) on the Illumina MiSeq platform. In addition to the three biological replicates described above, sequencing also included technical replicates generated from aliquots of libraries derived from two additional samples, to assess the technical variability of the method.

### 2.4. Ecological Analyses on Microbiota Data

Demultiplexed sequence reads were processed using QIIME2 [32] version 2024.2. Sequencing primers and reads without primers were removed using the Cutadapt tool [33]. DADA2 [34] was used to perform the filtering, merging, and chimera removal steps of the paired‐end reads after cutting low‐quality nucleotides from both forward and reverse reads. The taxonomic assignments were performed using the Scikit‐learn naive Bayes multinomial classifier re‐trained on the SILVA SSU database (Release 138) V3–V4 hypervariable database. All ASVs associated to genera with the mean relative abundance across samples below the 0.005% cut‐off were discarded to minimize contaminants and sequencing errors and to improve statistical inference [35, 36]. Every read identified as mitochondria or chloroplast sourced was discarded. Subsequently, the statistical analyses on bacterial communities were performed in R 4.3 with the help of the packages phyloseq 1.44.0 [37], vegan 2.6‐4, and other packages satisfying their dependencies. The packages ggplot2 3.4.2 and ggh4x 0.2.4 were used to plot data and results. A rarefaction analysis was performed on every sample at genus level using the function rarecurve (step 100 reads), further processed to highlight saturated samples (arbitrarily defined as samples with a final slope in the rarefaction curve with an increment in ASV number per reads < 1e‐4). The most abundant phyla and genera were identified according to their highest average relative abundance among the samples. The alpha‐diversity in each sample was estimated through observed richness, Shannon indices, and Pielou’s evenness indices. The function estimate_richness from phyloseq was used to compute the observed richness and Shannon values, while the Pielou’s evenness index was calculated using the formula *E* = *S*/log (*R*), where *S* is the Shannon diversity index and *R* is the observed ASV richness in the sample. Differences in alpha‐diversity indices were inspected using the Mann–Whitney test. PCoAs were performed using the Hellinger distance (Euclidean distance on square root proportions) on genera abundances to account for the sparse and compositional properties of the data [38]. Distances and dispersions were tested using PERMANOVA and Betadisper, respectively. Furthermore, significant differences in abundances of certain taxa between the groups were assessed using DESeq2 1.42.0 [39]. The resulting *p* values were adjusted with the Benjamini–Hochberg method. Therefore, every DESeq2 result with *p* value lower than 0.05 and log2FoldChange greater than 1 was considered significant. The statistical design deliberately did not account for subjects from the same household, as preliminary analyses indicated that this factor was not crucial in the current dataset and because of the uneven inclusion of control relatives across different patients. The MixOmics package [40] was employed to perform a sparse Partial Least Squares Discriminant Analysis (sPLS‐DA) on scaled proportional abundances using genera with a maximum percent abundance greater than 0.1 across samples. Three samples from both the CCHS and control groups were randomly selected and excluded from the sPLS‐DA model training to serve as a test dataset. A seed value of “1994” was manually set during these steps to maximize the outcome reproducibility. Further details regarding the sPLS‐DA model settings are provided in the R scripts.

The scripts used to process the 16S raw FASTQ and analyze the datasets are publicly available at the link https://github.com/LeandroD94/Papers/tree/main/2026_CCHS_Microbiota.

### 2.5. FAs and ACAs Extraction

The FAs and ACAs in the samples were analyzed in their free acid form using an isotopic dilution gas chromatography coupled with mass spectrometry (ID‐GC‐MS) method. The FAs and ACAs were extracted from the same frozen fecal samples used for DNA extraction. Specifically, two out of the 17 control samples collected did not contain sufficient remaining biomass to allow for FA and ACA analysis (see Table S1). After thawing, a buffer solution containing 50 mM NaHCO₃ and 200 mM Na₂CO₃ was added at a 1: 1 ratio (mg:*μ*L) to 250 mg of each sample in a 1.5‐mL centrifuge tube. The obtained suspension was briefly stirred in vortex apparatus, extracted in ultrasonic bath (for 5 min), and then centrifuged at 6000 g (for 10 min). The supernatant was collected and transferred in 1.5 mL centrifuge tube (sample solution). The FAs and ACAs were finally extracted as follows: an aliquot of 100 *μ*L of sample solution (corresponding to 100 mg of stool sample) was added of 50 *μ*L of internal standards mixture, 1 mL of tert‐butyl methyl ether, and 50 *μ*L of 6 M HCl solution in 1.5 mL centrifuge tube. Afterwards, each tube was shaken in vortex apparatus for 2 min, centrifuged at 10,000 rpm for 5 min, and finally the solvent layer was transferred in an autosampler vial and analyzed by ID‐GC‐MS method. All samples were prepared and processed three times by the method described above.

The following metabolites were quantified: SCFAs, such as acetic, propionic, isobutyric, butyric, isovaleric, 2‐methylbutyric, and valeric acids; MCFAs including isohexanoic, hexanoic, heptanoic, octanoic, nonanoic, and decanoic acids; and LCFAs such as dodecanoic, tetradecanoic, hexadecanoic, and octadecanoic acids. Additionally, the ACAs benzoic, phenylacetic, and phenylpropionic acids were quantified.

The analytes quantification through ID‐GC‐MS was performed according to the method previously described in [41]. The ionic signals used for the quantification of every analyte and its respective internal standard are reported in the Table S2.

### 2.6. FAs and ACAs Statistical Analyses

Statistical analyses of FA and ACA levels were performed on R 4.3 using their relative percentages. The Mann–Whitney *U* test was applied to assess differences between the CCHS and control groups. Subsequently, the significance of these differences was further assessed including also the age group as co‐factor in a linear model computed on ranks of the proportional quantities (i.e., rank (quantity) ~ age + group). Spearman’s rank correlation was used to evaluate associations between bacterial genera with a maximum percent abundance greater than 0.1 across samples and SCFA and ACAs.

Finally, the MixOmics package was employed to compute a sPLS‐DA on scaled proportional abundances of FAs. Three samples from the CCHS group and two from the control group were randomly selected and excluded from the sPLS‐DA model training to serve as a test dataset. A seed value of “1994” was manually set during these steps to stabilize the outcome reproducibility. The script used to analyze the FAs and ACAs, as well as further details regarding the related sPLS‐DA model settings, are provided in the R script available at the GitHub repository provided above.

## 3. Results

### 3.1. Preliminary Technical Assessment of the 16S Dataset

The first step of the 16S analysis involved performing a quality check on the obtained data. A total of 1,944,564 read pairs out of 3,546,835 (54.8%) passed the quality filtering steps. Of these, approximately 90% were successfully classified at the genus level. The samples’ ecological saturation was subsequently evaluated and confirmed by rarefaction curve analysis, indicating that the sequencing depth was sufficient for each sample (Figure S1). Preliminary PCoA including replicate samples revealed minimal biological variability and negligible technical variability, supporting dataset robustness (Figure S2). In particular, the replicate for sample “M1”, collected from a patient with CCHS, confirmed that its peculiar profile reflected a true biological condition rather than a technical artefact. As a result, no sample was identified as an outlier or failed quality assessment, and all were retained for downstream analyses. No samples were identified as outliers, and no clustering driven by subject age was observed (Figure S3).

### 3.2. Microbiota Profiling and Ecological Analyses

**Figure 1:**
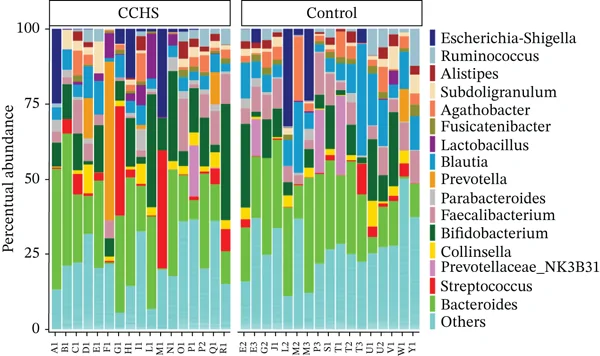
Barplot displaying the relative abundances of the most abundant genera in the dataset.

Microbiota profiles were analyzed starting with the most abundant taxa. Over 99% of the reads were classified as bacterial, with the remainder identified as archaeal. As expected, the most abundant phyla included Firmicutes (49.5%), Bacteroidota (30.7%), Actinobacteriota (13.9%), Proteobacteria (5.3%), and Verrucomicrobiota (0.3%). The 15 most abundant genera accounted for over 50% of the entire dataset and included *Escherichia-Shigella, Bacteroides*, *Streptococcus*, *Bifidobacterium*, *Collinsella*, *Faecalibacterium*, *Blautia*, among others (Figure 1). A complete list of the 15 most abundant genera and their relative abundances is provided in Table 1.

**Table 1:** Average percent abundances of the most abundant genera in the dataset.

Average across CCHS	Average across controls	Phylum	Genus
22.05	21.12	Bacteroidota	*Bacteroides*
13.83	8.78	Actinobacteriota	*Bifidobacterium*
4.93	10.88	Firmicutes	*Blautia*
5.01	6.8	Firmicutes	*Faecalibacterium*
4.73	4.02	Proteobacteria	*Escherichia-Shigella*
6.48	1.64	Firmicutes	*Streptococcus*
2.27	4.14	Firmicutes	*Agathobacter*
4.61	0.64	Bacteroidota	*Prevotella*
2.61	1.96	Actinobacteriota	*Collinsella*
1.03	3.5	Bacteroidota	*Prevotellaceae* NK3B31
1.81	2.62	Firmicutes	*Ruminococcus*
2.19	1.95	Firmicutes	*Subdoligranulum*
1.36	1.65	Bacteroidota	*Alistipes*
1.85	1.13	Bacteroidota	*Parabacteroides*
2.41	0.36	Firmicutes	*Lactobacillus*
0.93	1.48	Firmicutes	*Fusicatenibacter*

Although a few genera exhibited markedly different average relative abundances between CCHS and control groups, such as *Blautia* that was more abundant in the control group, these trends require dedicated statistical testing (performed below) for confirmation. The most abundant archaeal genus was *Methanobrevibacter*, with a mean abundance of 0.09% and substantial inter‐sample variability across both groups. Noteworthy, a few genera that are abundant and well‐documented in the literature, such as *Escherichia* and *Bifidobacterium*, exhibited high inter‐sample variability, sometimes being nearly undetectable even within the same group.

**Figure 2:**
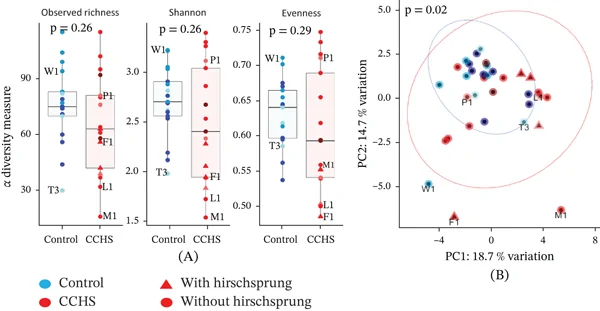
(A) Alpha diversity indices and (B) PCoA representing the beta diversity across samples. Control samples are represented in blue whereas the CCHS samples in red. The color shade (light blue to dark blue and light red to dark red) indicates the age of the subjects, with the lightest being the youngest. The triangles mark CCHS samples diagnosed also with Hirschsprung disease. Certain sample names, corresponding to outliers or peculiar subjects, are shown for selected points where necessary to facilitate the discussion.

Subsequently, the alpha and beta diversities were computed to assess intra‐sample and inter‐sample profile differences, respectively (Figure 2). No significant differences were detected in any alpha diversity indices (Figure 2A) or in beta dispersion between the CCHS and control groups (*B* *e* *t* *a* *D* *i* *s* *p* *e* *r* *Pr*(>*F*) = 0.17). In contrast, PERMANOVA analysis revealed a significant difference (*Pr*(>*F*) = 0.02) in overall taxonomic profiles between the two groups (Figure 2B). Notably, age alone was not a significant variable (*Pr*(>*F*) = 0.09), and the difference between CCHS and control groups consistently remained the only significant variable with all other factors losing significance (Table S3).

The difference between CCHS and control groups was less evident in the PCoA plot (Figure 2B), as the two groups did not form distinct clusters and most of the separation was driven by a few CCHS samples. Principal components (PC) 1 and 2 explained 18.7% and 14.7% of the total variance, respectively. Likewise, patients with CCHS who were also diagnosed with Hirschsprung disease did not display notable differences in microbiota composition compared with other patients with CCHS, according to the same analysis. However, two CCHS samples (labeled as M1 and F1 in Table S1 and in the paper figures), exhibited a notably distinct profile compared with other samples, positioning in the lower section of the PCoA plot. This result is coherent with the severity of their condition, which was already known at the time of recruitment. In details, both samples exhibited low alpha diversity: M1 showed the lowest observed richness, while F1 displayed low Pielou’s evenness, being predominantly colonized by *Prevotella* (Figure 1 and Figure 2A). Other CCHS samples did not share these characteristics or display a peculiar profile, including the sample L1, which had received a probiotic‐based treatment (see Materials and Methods) and clustered with the remaining samples. Among control, Subject W1, who had no known diseases, also displayed a distinct microbiota profile in the PCoA, likely attributable to the absence of detectable *Bifidobacterium* and *Alistipes* in the corresponding sample (Figure 1). Additionally, the youngest subjects in the dataset, control T3 (9 years old) and patient P1 with CCHS (11 years old), exhibited microbiota profiles comparable with older subjects. As an additional robustness check, the analysis was repeated using Bray–Curtis dissimilarity, which resulted in a highly similar pattern (Figure S3B).

### 3.3. Differential Abundance Analysis

**Figure 3:**
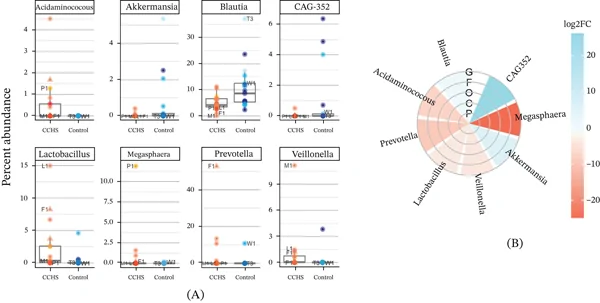
(A) Box plots displaying the relative abundances of taxa that are significantly differentially abundant between control subjects and patients with CCHS. Patients with CCHS diagnosed with Hirschsprung disease are indicated by triangular symbols. The color shade (light blue to dark blue and light red to dark red) indicates the age of the subjects, with the lightest being the youngest. Certain sample names, corresponding to outliers or peculiar subjects, are shown for selected points where necessary to facilitate the discussion. (B) CircoTax plot displaying log₂ fold change values, indicated through color intensities, of taxa that resulted significantly differentially abundant between control subjects and patients with CCHS.

Despite the overall small differences observed in PCoA, evident differences between CCHS and control groups were obtained when the relative abundances of each bacterium were analyzed (Figure 3).

The DESeq2 algorithm identified significantly higher abundances of Oscillospiraceae CAG‐352 (log2*F* *C* = 26, *p* *a* *d* *j* < 0.001), *Akkermansia* (log2*F* *C* = 6, *p* *a* *d* *j* < 0.001), and *Blautia* (log2*F* *C* = 1.5, *p* *a* *d* *j* = 0.02) in control subjects, whereas *Lactobacillus* (log2*F* *C* = −4.1, *p* *a* *d* *j* = 0.01), *Prevotella* (log2*F* *C* = −9.5, *p* *a* *d* *j* = 0.01), *Megasphaera* (log2*F* *C* = −24.7, *p* *a* *d* *j* < 0.001), *Veillonella* (log2*F* *C* = −4.4, *p* *a* *d* *j* = 0.005), and *Acidaminococcus* (log2*F* *C* = −7.6, *p* *a* *d* *j* = 0.02) were more abundant in the CCHS group (Figure 3). Of these, only *Blautia* and CAG‐352 remained significant if age was included in the model design. *Acidaminococcus*, *Lactobacillus*, and *Akkermansia* further presented distinct distributions across the two groups (Figure 3). *Megasphaera* and Oscillospiraceae CAG‐352 exhibited the largest absolute fold changes between the two groups (Figure 3B). The CCHS sample L1, which was receiving a probiotic supplement intended to promote the growth of *Lactobacillus*, exhibited the highest abundance of this genus in the dataset (Figure 3). Importantly, the significance of *Lactobacillus* was confirmed even after excluding this patient. Similarly, the peculiar M1 and F1 samples observed in the PCoA did not contribute to the observed results. Hirschsprung co‐morbidity in certain patients did not appear to underlie any of these results, based on the distributions of their abundances (Figure 3A).

**Figure 4:**
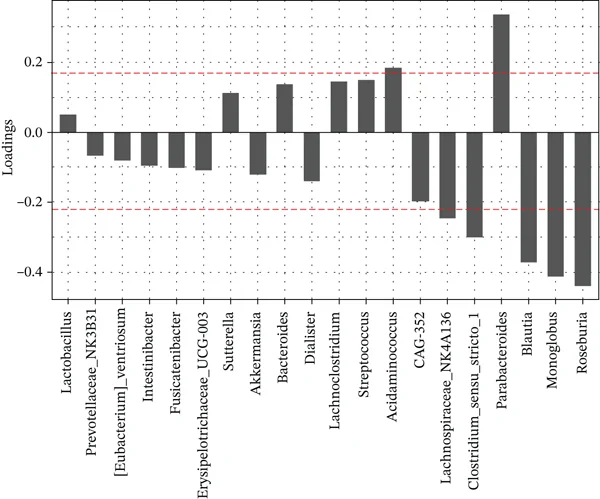
Bar plot displaying the loadings of the microbial genera that were selected on the first latent component in the related sPLS‐DA model. The red dotted lines are plot at 50% of both min and max loadings value.

To further explore group differences, a sPLS‐DA was performed (Figure S4). This supervised model effectively separated the two groups along the latent component (LC) 1 axis, selecting 20 genera on LC1 (Figure 4) and 1 genus on LC2 (Figure S4C) as interesting targets. *Roseburia* exhibited the highest absolute loading on LC1, followed by *Monoglobus* and *Blautia*, which displayed negative values (Figure 4). Additional genera, including *CAG-352*, *Akkermansia*, and *Eubacterium*, also showed negative loadings, whereas *Lactobacillus* and *Acidaminococcus* were associated with positive loadings. Notably, the sPLS‐DA loading patterns were consistent with most of the DESeq2 results, with features enriched in one group or the other showing loadings in opposite directions.

Despite sPLS‐DA substantially reinforced the differential analysis, the model did not exhibit a perfect classification performance, with an estimated Balanced Error Rate (BER) of approximately 35% if computed with two components (Figure S4B), correctly classifying four out of six test samples (using the “centroid distance” method). Nonetheless, several discriminant features identified by sPLS‐DA were consistent with those highlighted by DESeq2, supporting the robustness of the observed differential patterns.

### 3.4. Analyzing the FA and ACA Profiles

**Figure 5:**
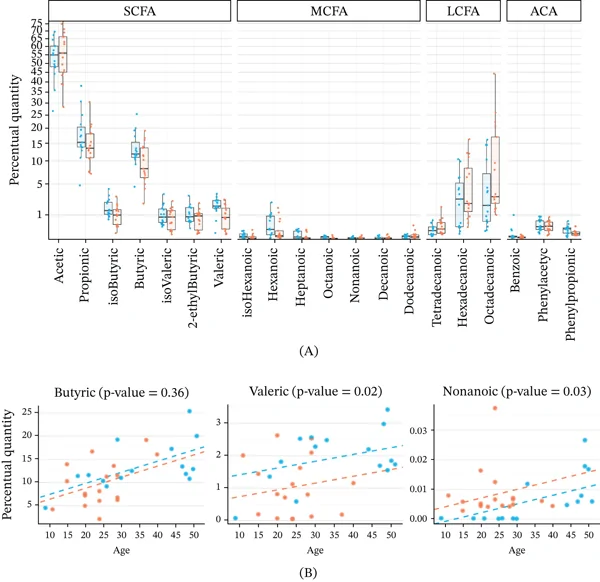
(A) Boxplots displaying the relative abundances of every FA and ACA analyzed across samples. (B) Simplified representations of the linear model computed on the ranked values of certain FAs (butyric, valeric, and nonanoic) including the age groups as co‐factor in the model design. The dotted lines represent the slopes and the intercepts of control or CCHS group for each of the represented FAs. The reported *p* values are related to the linear model including the co‐factors and are not adjusted. The quantities measured in control and CCHS subjects are colored in blue and red, respectively, in both A and B panels.

In parallel with microbiome profiling, the levels of fecal FA and ACA levels were assessed in CCHS and control groups (Figure 5).

Most FAs and ACAs did not display any notable differences between CCHS and control groups (Figure 5A). The SCFA acetic acid, propionic acid, and butyric acid were the most abundant across samples, with acetic acid being the predominant one overall (Figure 5A). As for the microbial investigation reported above, samples that exhibited a peculiar microbiota profile did not display any distinctive metabolite profile as well (Figure S5). The Mann–Whitney test revealed that valeric acid, isohexanoic acid, octanoic acid, benzoic acid, and, to a lesser extent, butyric acid were significantly higher in control group compared to the CCHS group (Figure S6). Notably, none of these differences remained significant after *p* value adjustment, likely due to the large number of statistical tests performed. Nevertheless, box plots show clearly visible differences in butyric, isohexanoic, octanoic, and valeric acid levels between groups (Figure S6). Additionally, if age is included as co‐factor in the linear model (Figure 5B), isohexanoic acid, valeric acid, and benzoic acid are confirmed significant. Butyric acid, while not significant, continued to show a slightly higher intercept in the case of the control group also in the model accounting for the samples’ age. Conversely, the nonanoic acid level was significantly higher in the CCHS group according to this model.

The LC1 of the sPLS‐DA model was further used to tentatively distinguish CCHS and control groups metabolite profiles (Figure S7A) resulting in a correct classification for four out of the five test samples, although with an estimated BER of the model during computation of approximately 40% (Figure S7B). Eight metabolites were chosen for LC1, with valeric, butyric, and octanoic acids displaying the highest loading values (Figure S7C). Propionic, nonanoic, and benzoic acids were selected for LC2 (Figure S7D), which played a smaller role in differentiating the two groups. Notably, only nonanoic acid exhibited a negative loading, in contrast to the other acids mentioned above, in agreement with the results obtained through univariate methods.

**Figure 6:**
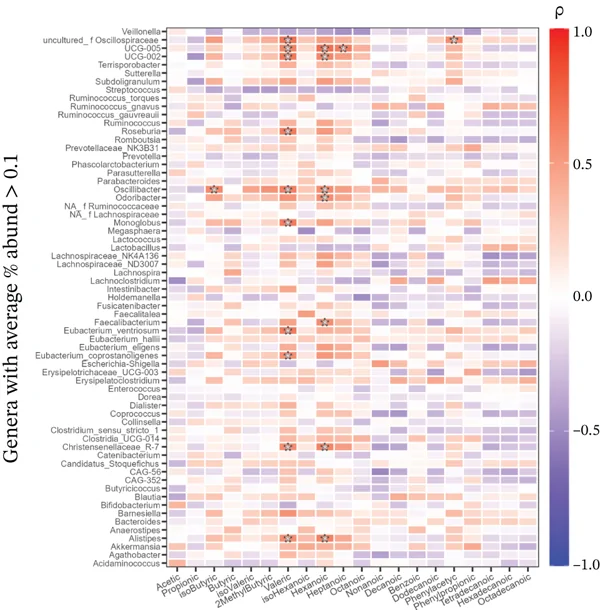
Heatmap of the spearman correlations between relative abundance of microbial genera with average abundance over 0.1% and relative quantities of FAs and ACAs. The “ ^∗^” symbol denotes the correlations remained significant after the *p* value adjustment.

Finally, the correlations between bacterial abundances and FA and ACA quantities were inspected (Figure 6, Table S4). After *p* value adjustment, positive correlations between valeric acid and various genera (including Oscillospiraceae UCG‐002, *Roseburia*, *Monoglobus, Oscillibacter*, and *Eubacterium*) were observed. Conversely, butyric, isohexanoic, and benzoic acids did not exhibit significant correlations.

## 4. Discussion

### 4.1. Microbiota Profiles Is Largely Conserved Across Patients and Controls

Despite our initial hypothesis that patients with CCHS might exhibit highly distinctive microbiota profiles, our results revealed only minor differences overall between the microbiota of patients with CCHS and controls. The dominant phyla and genera (Figure 1) identified across all samples were consistent with those typically reported in the healthy human gut microbiota in the existing literature [42, 43], including *Methanobrevibacter* as the predominant gut archaea [9]. Most patients with CCHS did not exhibit reduced alpha diversity values, contrarily to initial expectations based on the high level of ROS reported in this patients [5, 44, 45], resulting in comparable alpha diversity between the two groups (Figure 2A). Beta‐diversity analyses revealed a statistically significant difference in overall community profiles between CCHS and control groups. However, the effect size was small, as reflected in the PCoA (Figure 2B). Interestingly, no clear similarity patterns were observed among relatives belonging to the same household, sharing similar age or experiencing digestive issues. However, a larger cohort with more subjects from each of these potential sub‐groups and within the same sample group (i.e., representing both patients and control) is necessary to further discuss the impact of these co‐factors on microbiota composition.

### 4.2. Bacterial Biomarkers Linked to Inflammation and CCHS

Differential abundance analyses allowed the identification of discriminant genera between patients with CCHS and control groups. Overall, genera typically associated with a functionally eubiotic gut microbiota were more represented in controls, whereas patients with CCHS displayed higher abundances of genera frequently reported in contexts of intestinal inflammation. The role of each taxon’s abundance in discriminating between groups was evaluated using both a univariate unsupervised approach testing for differential abundance (Figure 3) and a multivariate supervised method selecting taxa based on the magnitude of their variation across samples (Figure 4). Importantly, the application of multiple algorithms aimed to highlight common outcomes by taking advantage of each method’s strengths while minimizing their respective weaknesses. This approach led us to focus primarily on the intersection of their results rather than on expanding the overall output.

Several genera were identified as significant by one or both approaches, including *Akkermansia, Blautia, Megasphaera, Monoglobus, Roseburia*, and *Eubacterium*, among others. Notably, these genera have been extensively associated in the literature with SCFA production. For instance, *Roseburia, Blautia, Monoglobus*, and *Eubacterium ventriosum* are SCFA producers, contributing to anti‐inflammatory processes and showing decreasing abundance in various diseases [23, 43, 46–48]. As stated above, we chose to focus the discussion on taxa that were consistently identified as significant by both approaches, thereby prioritizing precision over sensitivity. Accordingly, *Blautia* and Oscillospiraceae CAG‐352 (a family previously classified as *Ruminococcaceae* [49, 50]) emerged as the main distinguishing genera in control samples, whereas *Lactobacillus* and *Acidaminococcus* were more enriched in CCHS samples.

Both *Blautia* and Oscillospiraceae CAG‐352 are SCFA producers characteristic of “healthy” gut microbiota profile [51–53]. In addition, it is noteworthy that decreased abundances of *Blautia* and Oscillospiraceae are consistently observed in gut inflammatory diseases [53, 54]. These two genera were found to be robust regardless of the design used in the linear model, consistent with the well‐known positive effects they exert on the host. On the other hand, the interpretation of *Lactobacillus* and *Acidaminococcus* is less straightforward. *Acidaminococcus* is often reported to increase in various diseases [55, 56], including the inflammatory bowel diseases [57]. However, the species *Acidaminococcus intestini* thrives in the presence of acetate and is a butyrate producer [58, 59]. In light of this, we consider three scenarios as the most plausible: (i) other *Acidaminococcus* species increased in the CCHS group; (ii) *Acidaminococcus* shifts from a potentially beneficial to a detrimental one once a certain abundance is exceeded; or (iii) its enrichment represents an indirect consequence of an underlying dysbiosis. The *Lactobacillus* genus has been reported to exert either stimulatory or suppressive effects on Th1 lymphocyte activity and to induce both pro‐ and anti‐inflammatory mucosal cytokines, depending on the species involved [60–62]. Accordingly, drawing functional conclusions about *Lactobacillus* and *Acidaminococcus* role in host health is challenging given the taxonomic resolution limits of 16S amplicon sequencing [63, 64].

Nevertheless, we would like to emphasize a crucial yet often overlooked paradox in microbiota studies: the identification of statistically significant taxa, regardless of the robustness of the approach used, does not constitute direct evidence of their causal involvement in the disease process. These bacteria may simply reflect environmental selection pressures rather than act as primary drivers of the observed condition. This consideration is especially relevant in the context of CCHS, a syndrome driven by a well‐characterized genetic mutation. As such, our microbiota‐related observations could be regarded as downstream consequences of the patient’s underlying impairment. Once dysbiosis is established, a positive feedback loop occurs, with the bacteria further exacerbating the patient’s impairments, also leading to abnormal blood–barrier permeability and increased ROS levels [65]. In this scenario, our findings suggest the likelihood of inflammatory events in the gut of patients with CCHS, consistent with previous studies reporting elevated levels of several oxidative biomarkers in both their serum and urine [4, 5], which similarly point to inflammation. This is supported by the observation that co‐morbidity with Hirschsprung disease in a few subjects did not appear to influence our findings, corroborating the hypothesis that the primary driving factor underlying the microbiota differences was likely an intestinal inflammation, regardless of the specific disease involved.

### 4.3. FA and ACA Signatures in CCHS

In parallel with the gut microbiota analysis, fecal metabolomic profiling of SCFAs, MCFAs, LCFAs, and ACAs was conducted. The acetic acid, propionic acid, and butyric acid SCFAs were the FAs present in the highest quantities overall, displaying an approximate molar ratio of 3:1:1, in agreement with literature [11]. Most of the FAs and ACAs analyzed showed similar quantities between CCHS and control groups. However, the SCFAs butyric and valeric acids emerged as distinguishing features of the control subjects according to both univariate (Figure 5A, Figure S6) and multivariate methods (Figure S7), being present at lower levels across CCHS samples. Notably, butyric acid was not statistically significant in the univariate test, yet we still consider the observed difference noteworthy because of the distribution patterns and the *p* value (0.053) being very close to the conventional significance threshold (0.05). On the other hand, valeric acid was a far more robust result, being statistically confirmed regardless of the linear model design. Both butyric and valeric acids are known to exert positive effects on the host, being it reported as a natural product with anticancer, anti‐inflammatory, and immunomodulatory activity [12, 65, 66]. Valeric acid levels positively correlated with various genera harboring species capable of producing SCFAs, including Oscillospiraceae UCG‐002 (previously referred to as “Ruminoccoccaceae UCG‐002”), *Roseburia*, *Eubacterium ventriosum*, and *Oscillibacter* [23, 46, 49, 67]. In particular, *Oscillibacter valericigenes* is a taxon known to synthetize valeric acid [67], while *Eubacterium ventriosum* group has previously been correlated with valeric acid in literature [47]. These results support the hypothesis that, while not inducing a frank dysbiosis, CCHS may be associated with selective microbial and metabolic alterations potentially affecting gut functional balance. Nevertheless, although these correlations support the conclusion that variation in SCFAs producers is linked to variation in SCFAs in CCHS, correlation does not imply causation, as additional factors (e.g., other taxa, diet, and host genetics) may contribute to variation in FA or ACA levels. Accordingly, further studies are needed to confirm this biological association, including transcriptomic analyses and in vitro investigations.

Secondarily, the MCFAs octanoic (or “caprylic”) and nonanoic (or “pelargonic”) acids, as well as the ACA benzoic acid, were identified as significantly different, although they discriminated the CCHS group to a lesser extent than the above discussed SCFAs. Octanoic and nonanoic acids are substrates for oxidation to obtain energy [18] and may upregulate both the host antimicrobial peptide expression and the integrity of the intestinal epithelial barrier [68]. Conversely, the higher level of nonanoic acid was a distinguishing feature of CCHS in our observations, seemingly at odds with the beneficial role previously reported. On the other hand, benzoic acid is reported in the literature to mitigate inflammation, consistent with our findings showing higher levels of this ACA in the control group [69].

### 4.4. Overall Interpretation of Findings on CCHS and Comparison With OSAS

Taken together, the results of the present study indicate that CCHS is associated with gut microbiota and fecal metabolomic alterations that are overall modest in magnitude yet functionally coherent. The absence of a significant reduction in microbial alpha diversity, together with the limited separation observed in beta‐diversity analyses, suggests that CCHS is not characterized by a frank intestinal dysbiosis, but rather by selective changes in microbial composition and the associated metabolic output. This finding is particularly relevant, as it clarifies an aspect of CCHS biology that has not been previously addressed and helps to refine existing hypotheses regarding the role of the gut microbiota in this physiologically altered condition.

The enrichment of taxa typically associated with eubiotic, SCFA‐producing communities in control subjects, alongside the increased abundance in CCHS subjects of genera frequently reported in inflammatory contexts, points toward a functional shift of the gut ecosystem toward a less favorable inflammatory balance. This interpretation is further supported by fecal metabolomic data, which revealed reduced levels of SCFA with known anti‐inflammatory and trophic properties—most notably valeric acid and, to a lesser extent, butyric acid—in CCHS subjects. The integration of microbiota and metabolomic data therefore supports the hypothesis of a reduced anti‐inflammatory potential of microbial metabolism in CCHS, a biologically plausible scenario that is consistent with previous reports of increased oxidative stress in these patients [4, 5].

Emerging evidence suggests that gut‐derived metabolites such as SCFAs and ACAs may also influence systemic processes through the gut–brain and gut–lung axes, potentially modulating neuroimmune and respiratory functions. In CCHS, where PHOX2B gene mutations impair both central and enteric autonomic circuits, these alterations may have secondary effects beyond the gut, highlighting potential avenues for preventive or supportive strategies aimed at mitigating complications, although direct evidence in this population remains to be established.

Importantly, given the well‐defined genetic etiology of CCHS, the microbiota alterations observed in this study should not be interpreted as primary causal drivers of the disorder. Rather, they are more plausibly to be regarded as downstream functional consequences of the underlying pathophysiological condition and its long‐term management. Recognizing this distinction represents a meaningful conceptual advance, as it contributes to a more systemic understanding of CCHS and helps avoid misleading etiological interpretations of microbiota‐associated findings.

In this context, comparison with the existing literature on Obstructive Sleep Apnea Syndrome (OSAS) is particularly informative. It has been reported that OSAS—characterized by episodes associated with a reduction in blood oxygen saturation during sleep—is associated with more pronounced gut dysbiosis, often including reduced microbial diversity, more focused clustering of patient samples, enrichment of pro‐inflammatory taxa, and marked depletion of SCFA‐producing bacteria, in association with intermittent hypoxia, sleep fragmentation, and systemic inflammation [70–72]. In OSAS, gut microbiota alterations have been proposed to actively contribute to metabolic and cardiovascular comorbidities.

In contrast, the data obtained in patients with CCHS suggest a more selective and less severe impact on the gut microbial ecosystem. This difference may reflect the distinct physiologically altered features of CCHS, in which hypoventilation is chronic and often compensated by ventilatory support, rather than intermittent as in OSAS. Consequently, while the gut microbiota in OSAS may play a more active role in disease progression and systemic complications, the alterations observed in CCHS appear more consistent with adaptive or secondary functional changes.

### 4.5. Study Limitations

Despite its findings, this study presents certain limitations:

• The potential inflammatory interpretation is based on indirect evidence, including taxa associated with inflammatory diseases, reduced SCFA levels, and previous reports of increased ROS. However, further studies assessing inflammatory markers (e.g., calprotectin and serum IL‐6) are needed to assess this potential inflammatory state;
• All enrolled participants were of Italian origin, so different results (or similar results with varying magnitudes) might be observed in other ethnic groups, primarily due to differences in diets;
• A larger cohort, ideally including patients with Hirschsprung disease or other common comorbidities, would allow a better assessment of their potential impact on the CCHS‐associated microbiota;
• Our statistical approach was intentionally stringent to ensure focusing on the most robust results, but alternative analytical strategies might prefer to minimize false negatives rather than false positives.

To help address these limitations and promote transparency, both raw and processed data from this study have been made publicly available (see Data Availability Statement).

## 5. Conclusions

This study provides the first integrated characterization of gut microbiota composition and fecal metabolomic profiles in patients with CCHS, revealing subtle but biologically relevant alterations likely reflecting functional consequences of the disorder rather than primary causal factors.

Although mechanical ventilation remains the cornerstone of CCHS management, our findings highlight that CCHS should not be regarded solely as a respiratory disorder. Rather, it manifests as a systemic condition, leaving distinctive gut microbiota and metabolomic signatures that reflect documented oxidative stress and potential secondary metabolic and inflammatory alterations, the latter inferred from microbial composition and SCFA profiles. These signatures can be considered fingerprints associated with the CCHS syndrome.

The analysis highlights functional shifts in gut microbiota and associated metabolites, including SCFAs such as valeric and butyric acid, known for their anti‐inflammatory and immunomodulatory properties. Specific microbial taxa, such as *Blautia* and Oscillospiraceae CAG‐352 in control subjects and *Lactobacillus* and *Acidaminococcus* in patients with CCHS, further suggest a shift toward a less favorable inflammatory balance. While these findings are not immediately translatable into validated clinical biomarkers, they provide exploratory multi‐omics functional signatures that can guide future mechanistic studies and inform personalized supportive strategies, including nutritional interventions and microbiota‐targeted approaches, aimed at supporting gut and systemic homeostasis. Importantly, studies profiling gut microbiota in rare genetic diseases with a defined causative gene are extremely limited. Among such conditions, only cystic fibrosis and CDKL5 deficiency have been explored, making comparative data scarce. This underscores the novelty and relevance of our work in CCHS and highlights the value of meticulous sample collection and integrated multi‐omics analyses. By providing the first functional insight into microbiota–host interactions in this genetically defined rare disorder, our study lays a translational foundation for understanding secondary, potentially modifiable processes that contribute to disease burden. Targeted investigations incorporating gut inflammatory markers, as well as coordinated multicenter efforts to expand cohort sizes will be essential to validate these observations and explore their potential utility in optimizing long‐term patient supportive care.

Overall, this work represents a meaningful step forward in understanding of CCHS, providing a rational foundation for future studies aimed at improving supportive care and long‐term health outcomes in this rare patient population.

## Author Contributions

Conceptualization, data management and supervision: Matteo Ramazzotti and Donatella Degl’Innocenti; amplification and data analysis: Leandro Di Gloria; sample extraction, preparation and analysis: Marzia Vasarri; SCFA analysis: Marta Menicatti and Gianluca Bartolucci; Resources, Data curation: Niccolò Nassi, Marta Peruzzi, Monica Annunziata, Arianna Bedeschi; funding acquisition: Donatella Degl’Innocenti; Matteo Ramazzotti and Donatella Degl’Innocenti jointly supervised this work. Donatella Degl’Innocenti and Niccolò Nassi are joint last co‐authors.

## Funding

This study was supported by A.I.S.I.C.C. and University of Florence (Fondi di Ateneo 2025). Open access publishing facilitated by Universita degli Studi di Firenze, as part of the Wiley ‐ CRUI‐CARE agreement.

## Disclosure

All authors have read and approved the published version of the manuscript.

## Ethics Statement

Ethical approval for this study was obtained from Ethics Committee of the Region of Tuscany – Pediatrics, May 3, 2024, n° 57/2024, and all participants or their legal guardians provided written informed consent in accordance with the Declaration of Helsinki.

## Conflicts of Interest

The authors declare no conflicts of interest.

## Supporting information


**Supporting Information** Additional supporting information can be found online in the Supporting Information section. Figure S1: Rarefaction curves of the samples displaying the respective ecological saturation at genus level. The number of reads (after every quality filter) is reported on the X axis, while the number of genera is indicated on the Y axis. Figure S2: PCoA computed on genera percentages using the Hellinger distance and including the sample replicates. Control samples are represented in blue whereas the CCHS samples in red. Lines connect the replicates from the same sample. Ellipses are drawn around CCHS and Control group centroids, representing the 95% confidence interval. The name of each sample (see Table 1) is reported on each point. Figure S3: (A) PCoA computed on genera percentages using the Hellinger distance and representing the beta diversity across samples, with focus on the sample age. Control samples are represented as circles whereas the CCHS samples are triangles. The color shade (light blue to dark blue and light red to dark red) indicates the age of the subjects, with the lightest being the youngest. The age of the sample is written on the respective point. (B) PCoA based on Bray–Curtis dissimilarity calculated from genus‐level proportional abundances. Certain sample names, corresponding to outliers or peculiar subjects, are shown for selected points where necessary to facilitate the discussion. Figure S4: (A) Sparse PLS‐DA computed on scaled proportional abundances of microbial genera. (B) BER (Balanced error rate) for each component in the sparse PLS‐DA. (C) Loadings of the selected genera on latent component 2 (LC2). Figure S5: PCoA computed on metabolite percent quantities using the Euclidean distance and representing the diversity in metabolite profiles across samples, with focus on the sample age. Control samples are represented in blue whereas the CCHS samples in red. The sample name is written on the respective point. Figure S6: Box plots of the percent quantities of the metabolites significantly different between control and CCHS groups according to the Mann–Whitney test. The related *p* values are reported on the top of each box plot. Figure S7: (A) Sparse PLS‐DA computed on scaled proportional quantities of FAs and ACAs. (B) BER (Balanced error rate) for each component in the sparse PLS‐DA. Loadings of the selected FAs and ACAs on latent component 1 (C) and latent components 2 (D). Table S1: Table containing information for each sample analyzed. The “household group” column indicates whether samples come from the same household, with samples from the same household marked by the same letter. The “16S analysis” and “metabolite analysis” columns indicates, respectively, if the sample microbiota and metabolites were analyzed. The “16S replicate” indicates if the sample featured a replicate during the preliminary microbiota analysis. The “Hirschprung disease” column reports if the CCHS is diagnosed with Hirschsprung. Table S2: Retention times (Rt) and ionic signals used for quali‐quantitation of SCFAs and relative internal standards used. Table S3: *p* values of the difference between CCHS and control group profiles whereas the PERMANOVA includes different co‐factors. Table S4: Rho and *p* values of the spearman correlations between genera abundances and FAs quantities.

## Data Availability

The raw sequencing data is publicly available on NCBI SRA (Sequence Read Archive) at bio‐project PRJNA1412517. The raw metabolite tables and the analysis scripts are available on GitHub: https://github.com/LeandroD94/Papers/tree/main/2026_CCHS_Microbiota.
