# Supplementary material for: Microbiota–Metabolome Alterations Suggest Pro‐Inflammatory Phenotype in Congenital Central Hypoventilation Syndrome
Source: Int J Microbiol. 2026 Sep 7;2026:7551500. doi: 10.1155/ijm/7551500 (PMC13550528; doi:10.1155/ijm/7551500)
Supplement: Supplementary file 1 [file IJM-2026-7551500-s001.zip › Supplementary files and captions _ REVISED.pdf]

SUPPLEMENTARY FIGURES

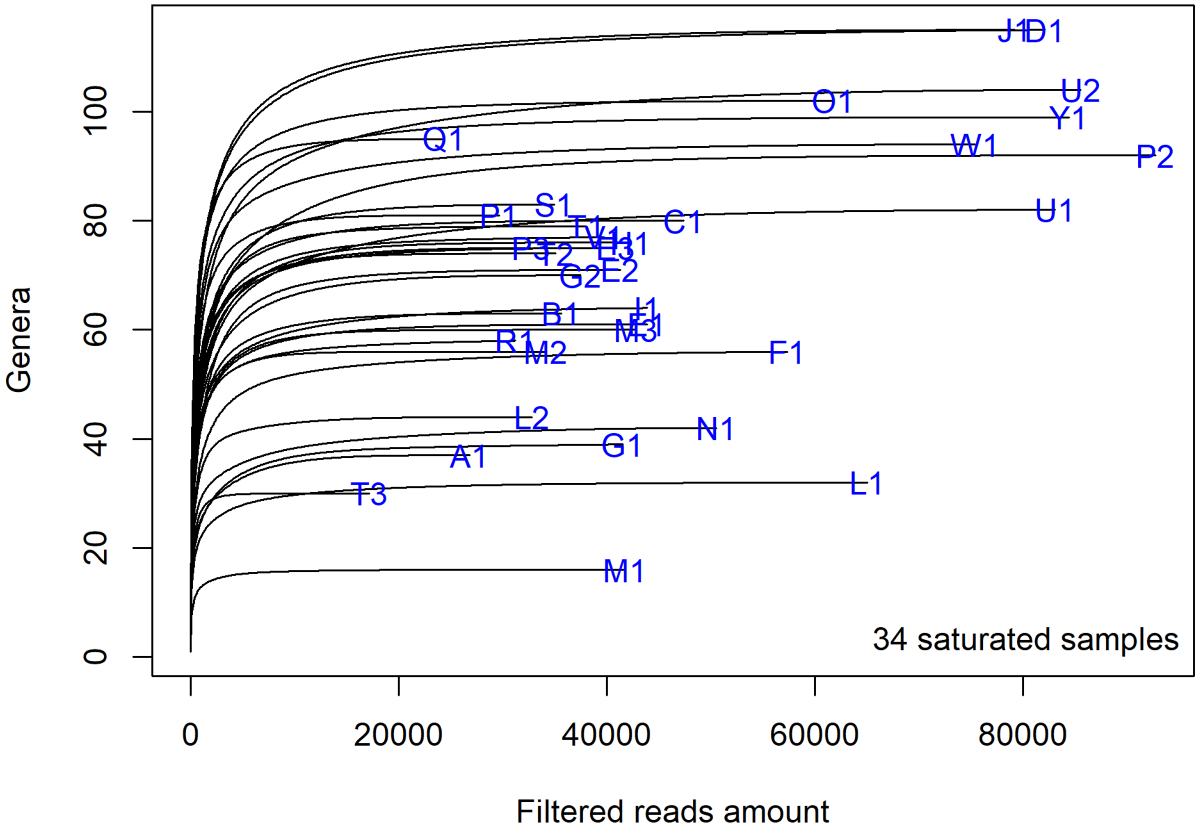

Supplementary figure 1: Rarefaction curves of the samples displaying the respective ecological saturation at genus level. The number of reads (after every quality filter) is reported on the X axis, while the number of genera is indicated on the Y axis.

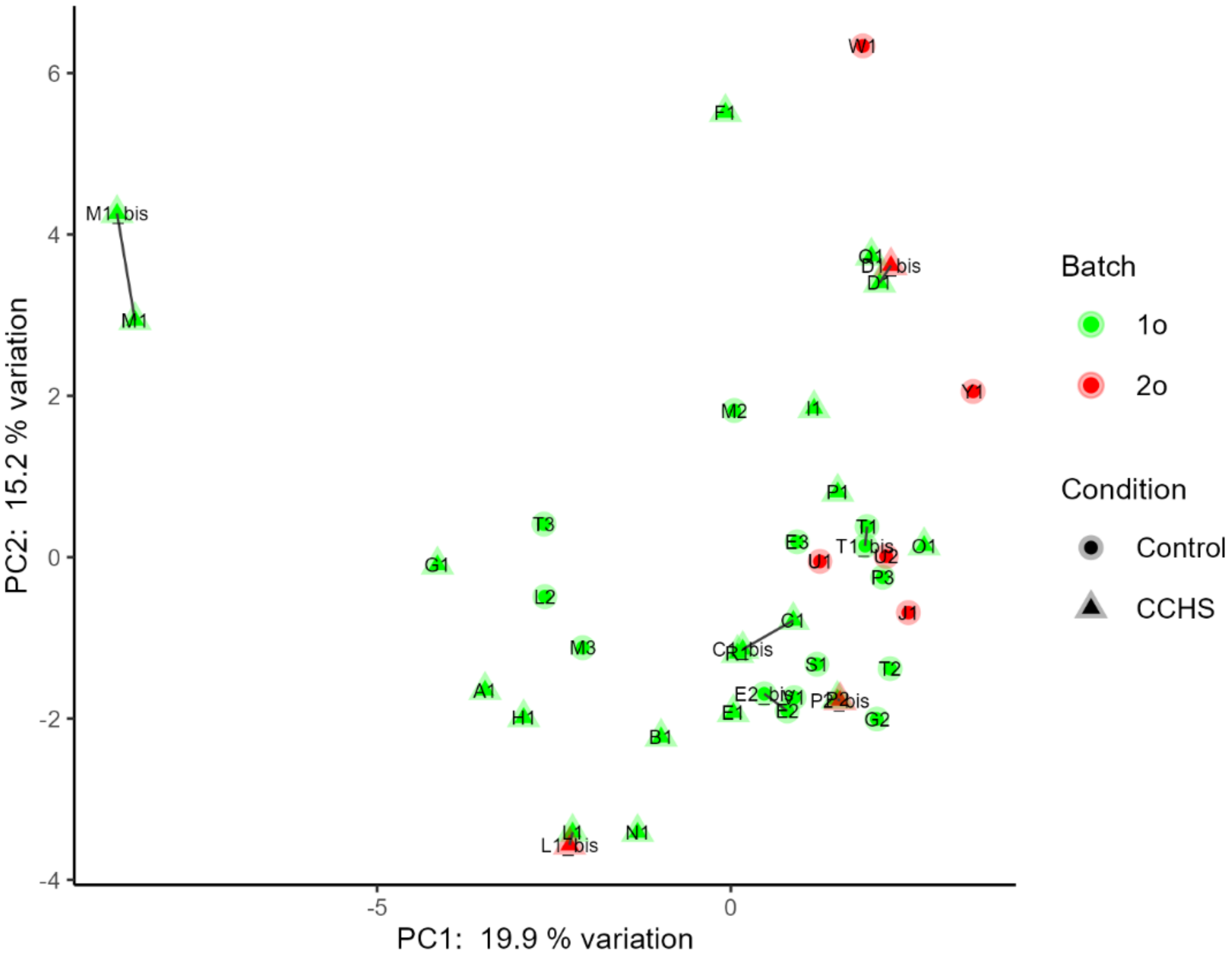

Supplementary figure 2: PCoA computed on genera percentages using the Hellinger distance and including the sample replicates. Control samples are represented in blue whereas the CCHS samples in red. Lines connect the replicates from the same sample. Ellipses are drawn around CCHS and Control group centroids, representing the 95% confidence interval. The name of each sample (see table 1) is reported on each point.

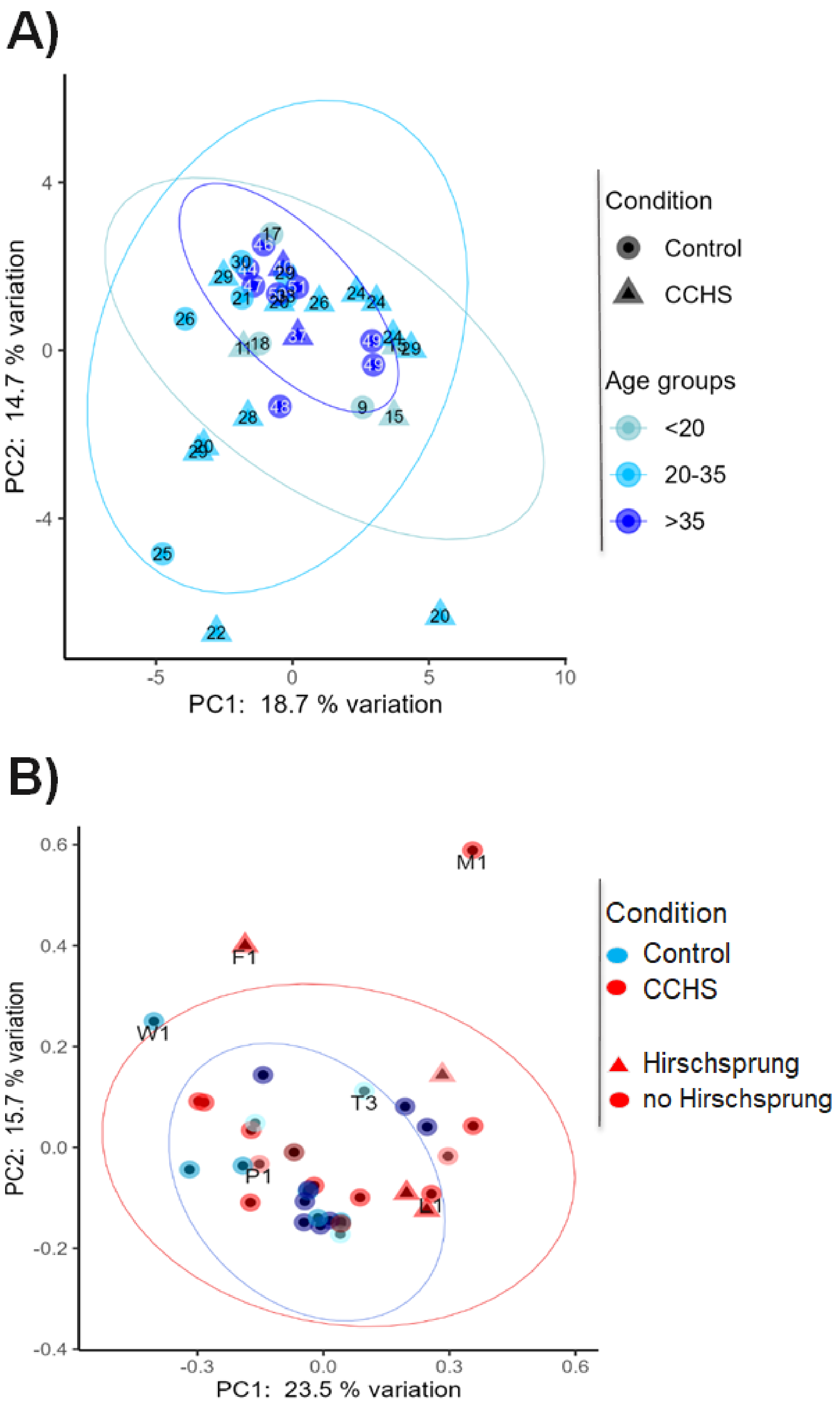

Supplementary figure 3: A) PCoA computed on genera percentages using the Hellinger distance and representing the beta diversity across samples, with focus on the sample age. Control samples are represented as circles whereas the CCHS samples are triangles. The colour shade (light blue to dark blue and light red to dark red) indicates the age of the subjects, with the lightest being the youngest. The age of the sample is written on the respective point. B) PCoA based on Bray–Curtis dissimilarity calculated from genus-level proportional abundances. Certain sample names, corresponding to outliers or peculiar subjects, are shown for selected points where necessary to facilitate the discussion.

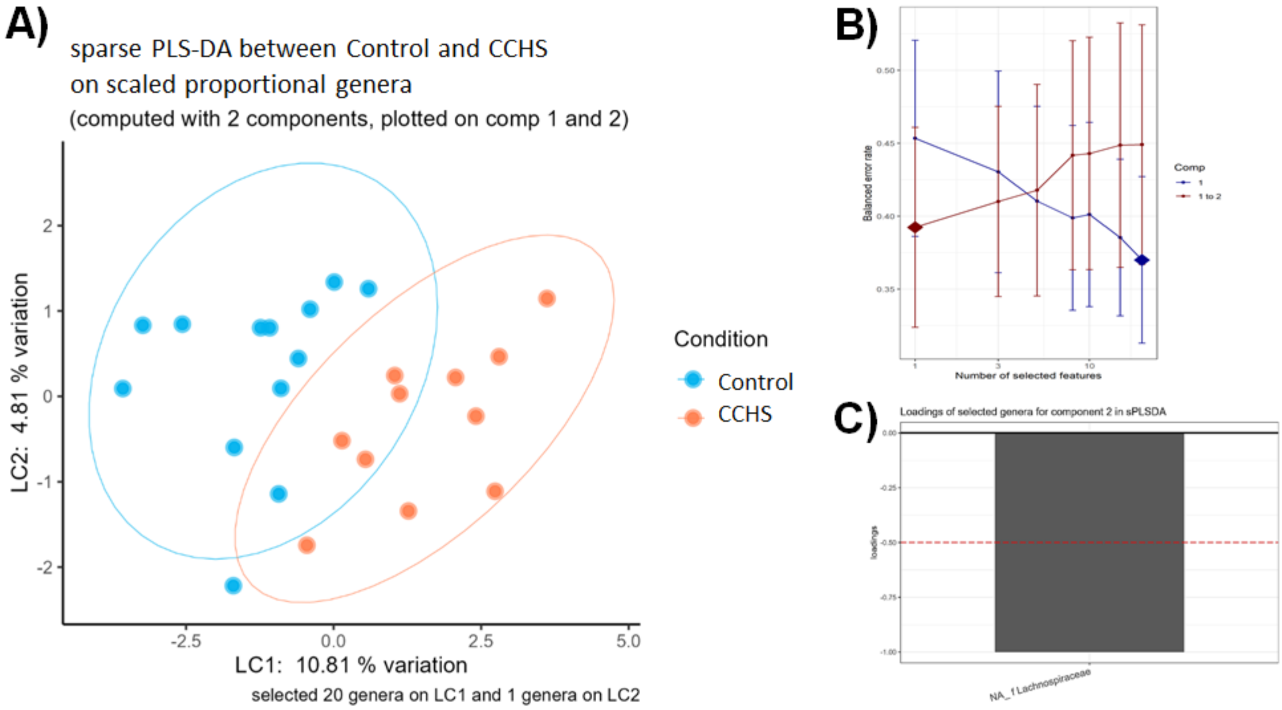

Supplementary figure 4: A) Sparse PLS-DA computed on scaled proportional abundances of microbial genera. B) BER (Balanced error rate) for each component in the sparse PLS-DA. C) Loadings of the selected genera on latent component 2 (LC2).

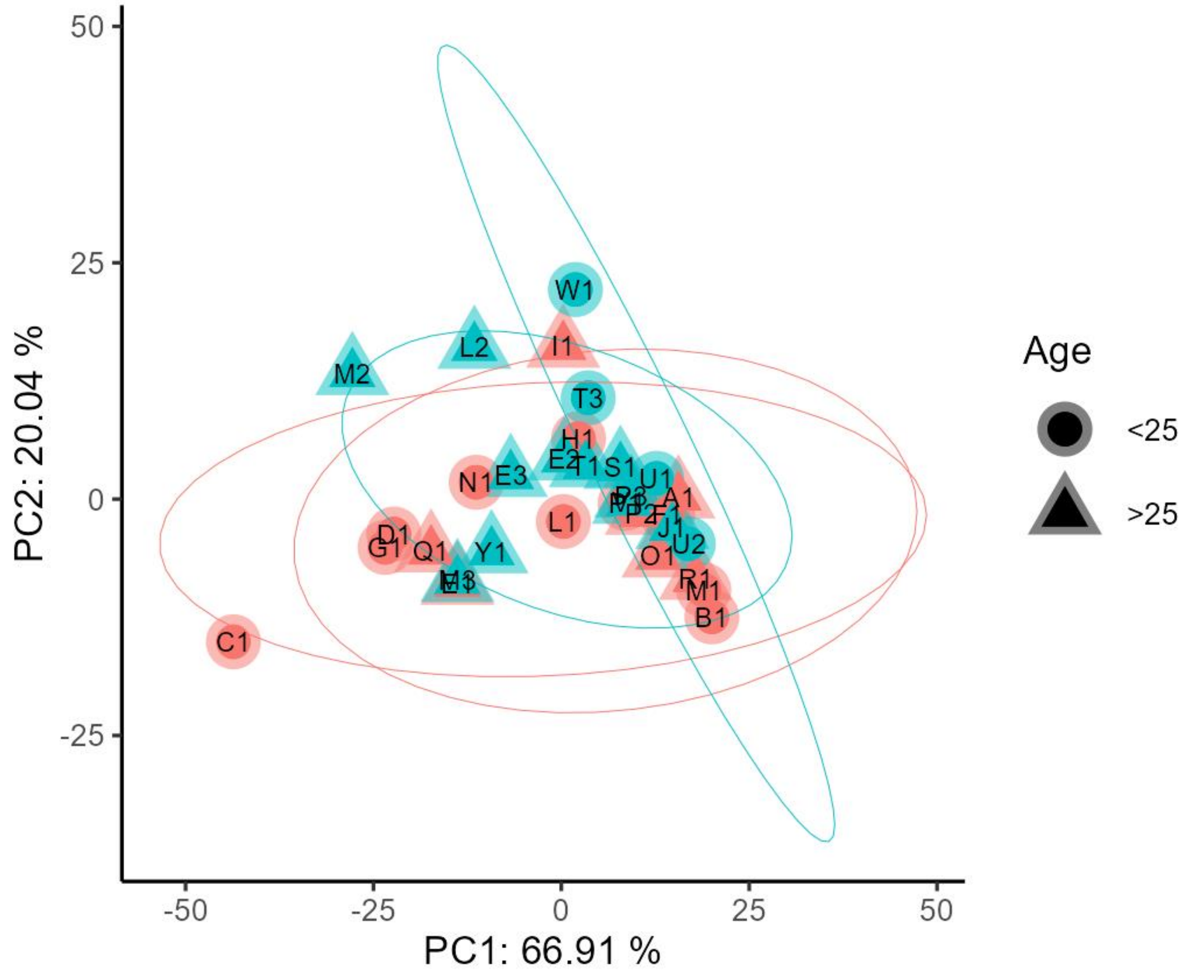

Supplementary figure 5: PCoA computed on metabolite percent quantities using the euclidean distance and representing the diversity in metabolite profiles across samples, with focus on the sample age. Control samples are represented in blue whereas the CCHS samples in red. The sample name is written on the respective point.

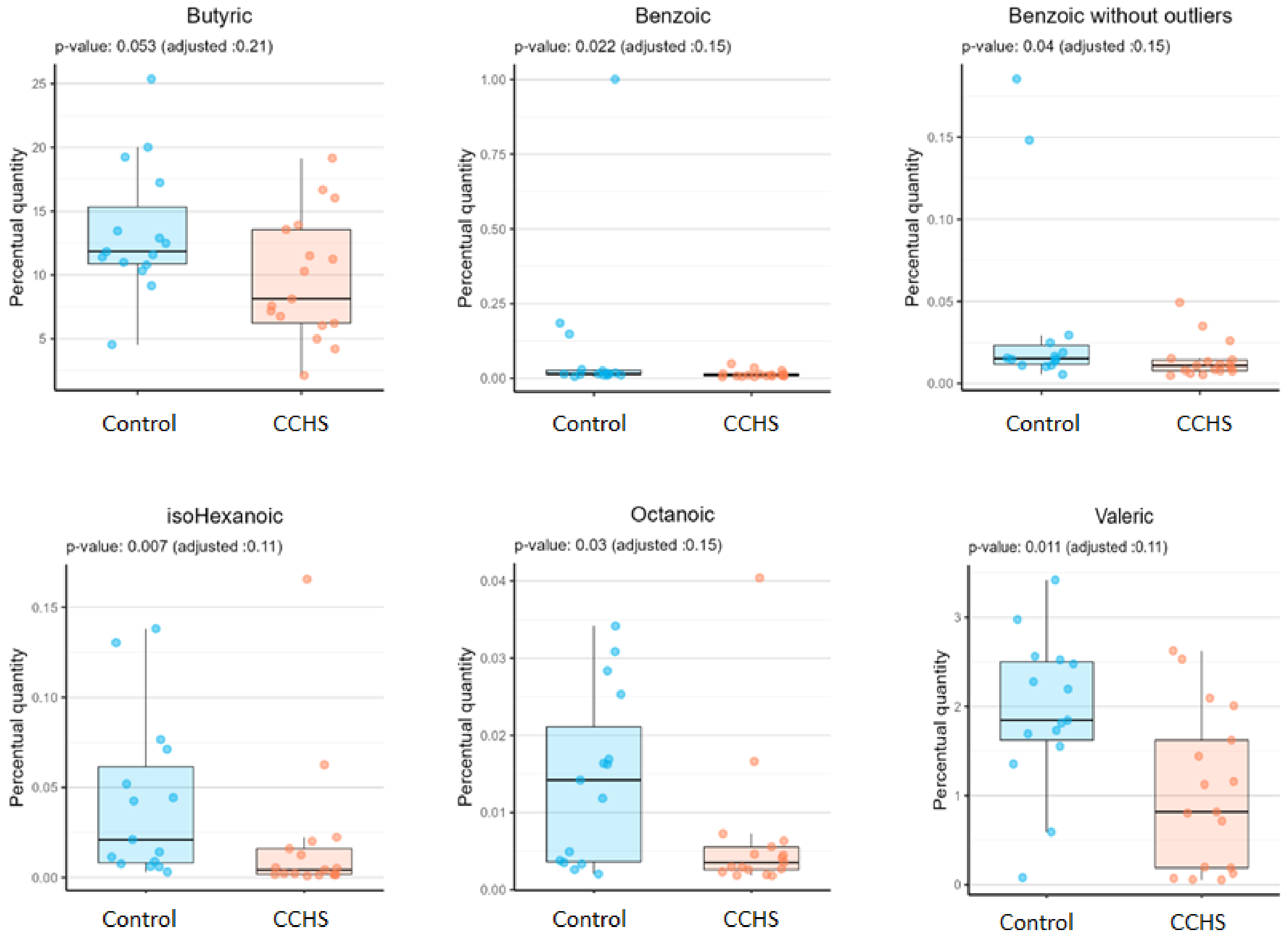

Supplementary figure 6: Box plots of the percent quantities of the metabolites significantly different between control and CCHS groups according to the Mann-Whitney test. The related p-values are reported on the top of each box plot.

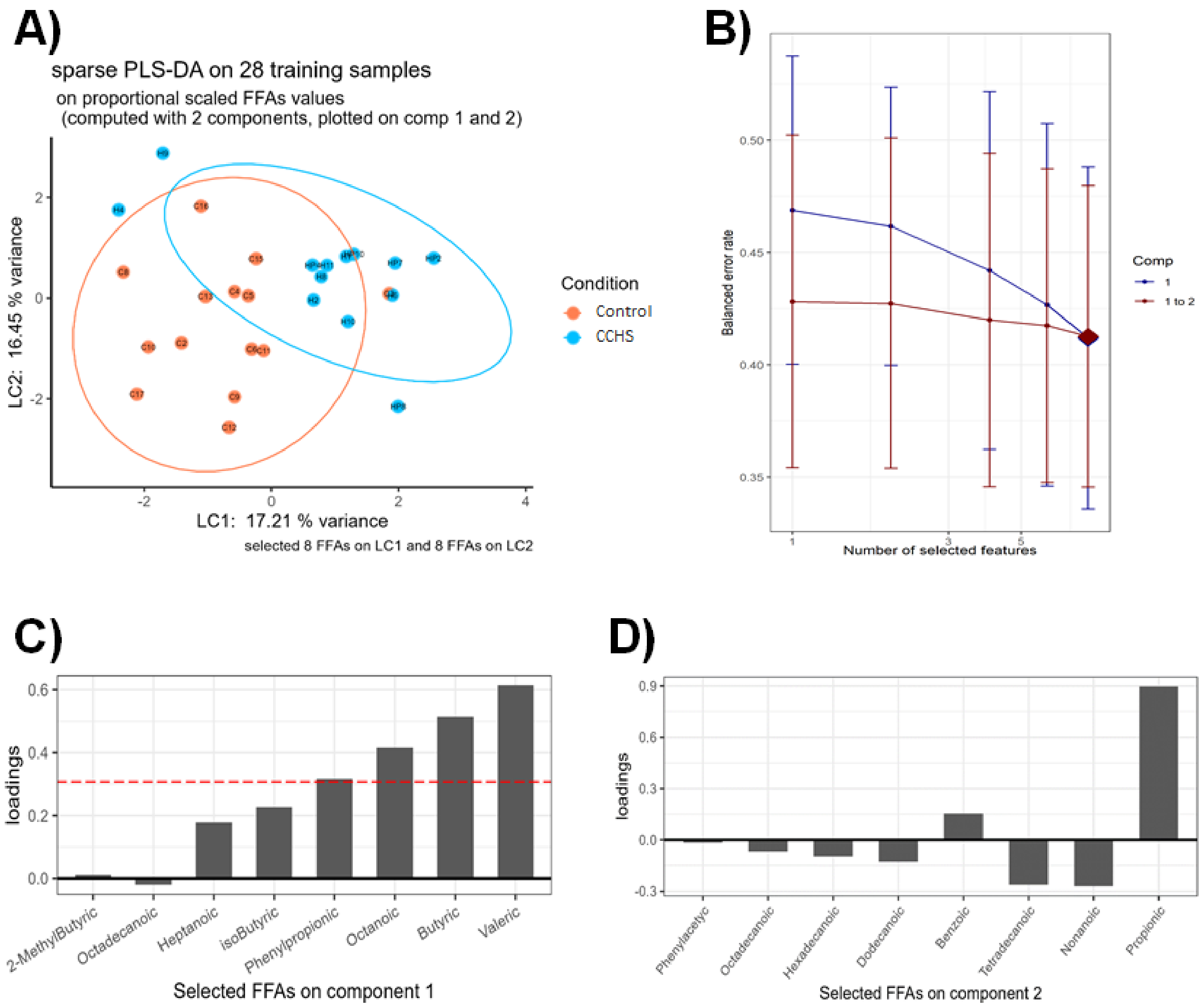

Supplementary figure 7: A) Sparse PLS-DA computed on scaled proportional quantities of FAs and ACAs. B) BER (Balanced error rate) for each component in the sparse PLS-DA. Loadings of the selected FAs and ACAs on latent component 1 (C) and latent components 2 (D).

SUPPLEMENTARY TABLES

Sample	Condition group	Household group	Age	Sex	Hirschsprung disease	16S analysis	16S replicate	Metabolites analysis
A1	CCHS	A	29	F	No	Yes	No	Yes
B1	CCHS	B	24	F	Yes	Yes	No	Yes
C1	CCHS	C	20	M	No	Yes	Biological	Yes
D1	CCHS	D	20	F	No	Yes	Technical	Yes
E1	CCHS	E	26	M	No	Yes	No	Yes
F1	CCHS	F	22	M	Yes	Yes	No	Yes
G1	CCHS	G	15	M	Yes	Yes	No	Yes
H1	CCHS	H	15	M	No	Yes	No	Yes
I1	CCHS	I	28	M	No	Yes	No	Yes
L1	CCHS	L	24	F	No	Yes	Technical	Yes
M1	CCHS	M	20	F	No	Yes	Biological	Yes
N1	CCHS	N	24	M	Yes	Yes	No	Yes
O1	CCHS	O	29	F	No	Yes	No	Yes
P1	CCHS	P	11	F	No	Yes	No	Yes
P2	CCHS	P	40	F	No	Yes	No	Yes
Q1	CCHS	Q	29	M	No	Yes	No	Yes
R1	CCHS	R	37	M	No	Yes	No	Yes
E2	Control	E	51	F	No	Yes	Biological	Yes
E3	Control	E	50	M	No	Yes	No	Yes
G2	Control	G	17	F	No	Yes	No	No
J1	Control	J	30	M	No	Yes	No	Yes
L2	Control	L	49	M	No	Yes	No	Yes
M2	Control	M	48	F	No	Yes	No	Yes
M3	Control	M	49	M	No	Yes	No	Yes
P3	Control	P	44	M	No	Yes	No	Yes
S1	Control	S	29	M	No	Yes	No	Yes
T1	Control	T	47	M	No	Yes	Technical	Yes
T2	Control	T	46	F	No	Yes	No	No
T3	Control	T	9	F	No	Yes	No	Yes
U1	Control	U	18	F	No	Yes	No	Yes
U2	Control	U	21	F	No	Yes	No	Yes
V1	Control	V	33	F	No	Yes	No	No
W1	Control	W	25	M	No	Yes	No	Yes
Y1	Control	Y	26	F	No	Yes	No	Yes

Supplementary table 1: Table containing information for each sample analysed. The “household group” column indicates whether samples come from the same household, with samples from the same household marked by the same letter. The “16S analysis” and “metabolite analysis” columns indicates, respectively, if the sample microbiota and metabolites were analysed. The “16S replicate” indicates if the sample featured a replicate during the preliminary microbiota analysis. The “Hirschprung disease” column reports if the CCHS is diagnosed with Hirschsprung.

Acids	Rt (min)	Quan. Ion (m/z)	Qual. Ion (m/z)	Internal standards (ISTDs) [Quan. ion (m/z)]
Acetic	4.78	60	43	[$^2H_3$]Acetic [63]
Propionic	5.17	74	73	[$^2H_5$]Propionic [77]
iso-Butyric	5.28	73	88	[$^2H_7$]iso-Butyric [77]
Butyric	5.54	60	73	[$^2H_7$]Butyric [63]
iso-Valeric	5.72	60	73	[$^2H_9$]iso-Valeric [63]
2-Methylbutyric	5.75	74	73	[$^2H_9$]iso-Valeric [63]
Valeric	5.98	60	73	[$^2H_9$]Valeric [63]
Iso-Hexanoic	6.26	74	73	[$^2H_{11}$]Hexanoic [63]
Hexanoic	6.42	60	73	[$^2H_{11}$]Hexanoic [63]
Heptanoic	6.8	60	73	[$^2H_{11}$]Hexanoic [63]
Octanoic	7.17	60	73	[$^2H_{15}$]Octanoic [63]
Nonanoic	7.53	60	73	[$^2H_{15}$]Octanoic [63]
Decanoic	7.88	60	73	[$^2H_{15}$]Octanoic [63]
Benzoic	8.46	105	77	[$^2H_{15}$]Octanoic [63]
Dodecanoic	8.62	60	73	[$^2H_{15}$]Octanoic [63]
PhenylAcetic	8.9	91	136	[$^2H_9$]Valeric [63]
PhenylPropionic	9.19	91	136	[$^2H_9$]Valeric [63]
Tetradecanoic	9.56	60	73	[$^2H_{31}$]Hexadecanoic [63]
Hexadecanoic	10.9	60	73	[$^2H_{31}$]Hexadecanoic [63]
Octadecanoic	12.97	60	73	[$^2H_{31}$]Hexadecanoic [63]

Supplementary table 2: Retention times (Rt) and ionic signals used for quali-quantitation of SCFAs and relative internal standards used.

* *Due to its large size, Table S3 is not included in this document and is instead provided as a separate file*

Supplementary table 3: P-values of the difference between CCHS and control group profiles whereas the PERMANOVA includes different co-factors.

* *Due to its large size, Table S4 is not included in this document and is instead provided as a separate file*

Supplementary table 4: Rho and p-values of the spearman correlations between genera abundances and FAs quantities.
